# Identification of three master regulatory genes with prognostic value for uveal melanoma by means of weighted co‐expression network analysis

**DOI:** 10.1111/srt.13358

**Published:** 2023-07-03

**Authors:** Michael Joseph Diaz, Jasmine Thuy Tran, Marjorie Montanez‐Wiscovich

**Affiliations:** ^1^ College of Medicine University of Florida Gainesville Florida USA; ^2^ School of Medicine University of Indiana Indianapolis Indiana USA; ^3^ Department of Dermatology University of Florida Gainesville Florida USA

1

To the Editor,

Uveal melanoma (UM) is a rare intraocular cancer of the uvea or uveal tract, usually implicating a GNAQ or GNA11 mutation.^1^ Patient outcomes are poor, with probability of metastasis above 50% and subsequent low long‐term survival rates.^2^ Based on epidemiologic estimates are equally sobering: UM represents 5% of all primary melanoma diagnoses and has a mean‐age‐adjusted incidence > 5 cases per million individuals per year in the United States.^3^ Prior expression‐based profiling efforts have revealed potential predictors of metastatic progression,^4^, ^5^ but this has yet translated to clinical benefits. The aim of this study is to elucidate regulatory genes that are associated with patient outcomes, so as to identify new targets for future UM therapies.

A weighted gene co‐expression network analysis was performed to identify candidate hub genes. Bulk RNA sequencing data and corresponding survival information were sourced from the Broad GDAC Firehose portal (cohort = UVM) (*N* = 80). Genes were retained for downstream analysis if they (1) were among the 80th percentile by variance, (2) were represented by >4 nonmissing samples, and (3) had a calculated median absolute deviation >0. Samples were clustered hierarchically (method = unweighted pair group method with arithmetic mean) to identify outliers. The analyzed expression matrix represented 78 samples and 3839 genes. Counts subjected to transcripts per million normalization and log2‐transformation were used for downstream analysis. Gene co‐expression analysis was performed with R package “WGCNA” v1.71 (networkType = “signed,” corType = “bicor,” power = 18, minModuleSize = 20, deepSplit = 4, maxPOutliers = 0.1).^6^ The soft thresholding power was determined by consulting the scale free topology (Figure 1). Hub genes were defined as the most connected gene (i.e., highest correlation) within each candidate module. Kaplan–Meier analysis of overall and disease‐free survival (OS, DFS) outcomes was conducted using R package “survminer.” For each hub gene, UM patients with above‐median gene expression were compared to UM patients with below‐median gene expression. *p*‐Values less than 0.05 were considered statistically significant.

**FIGURE 1:**
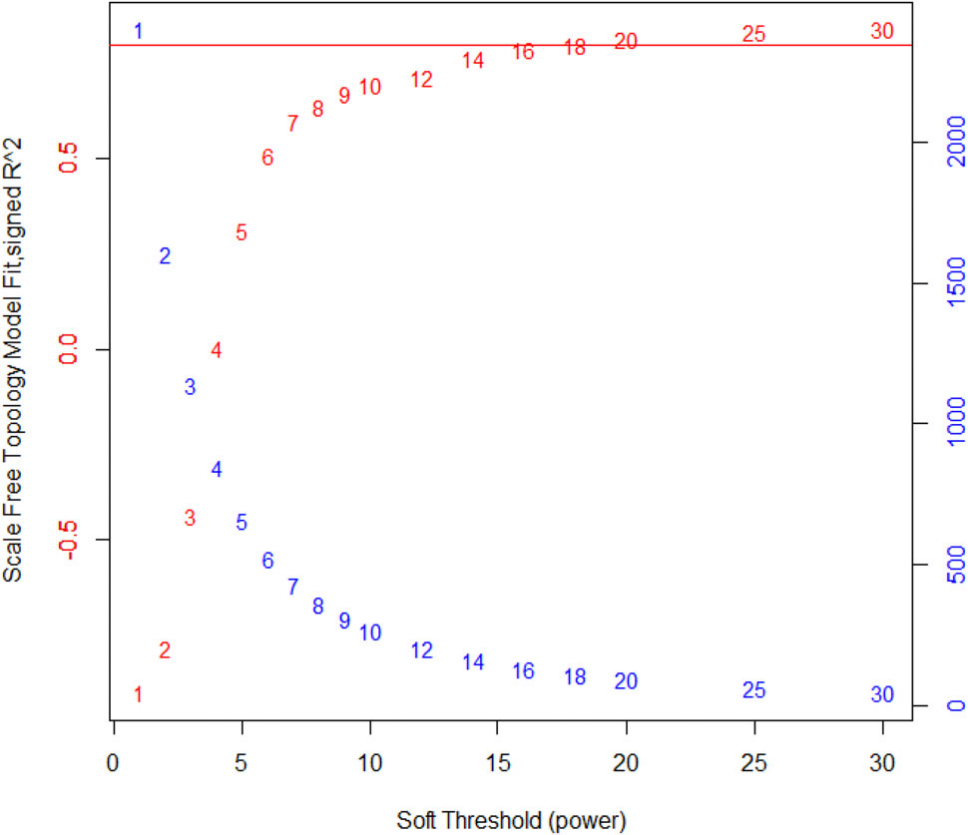
Analysis of network scale independence and mean connectivity at soft‐thresholding powers 1–10 stepwise, 12–20 incrementing by 2, 25, and 30. Relevant parameters were networkType = “signed,” verbose = 5, corFnc = “bicor,” and corOptions = list(use = ‘p’, maxPOutliers = 0.1). Red line demarcates signed R^2 of 0.8.

A total of six regulatory networks were generated. Lower expression of SASH3 (*p*‐value = 0.0011) and HM13 (*p*‐value = 0.0001) correlated with better OS outcomes. Higher expression of PLXNB1 correlated with better OS and DFS outcomes (*p*‐value < 0.0001). DFS analysis of HM13 and PLXNB1 revealed similarly significant trends. Table 1 has a comprehensive report of these gene‐survival associations. Prior Pan‐Cancer analysis linked high expression of HM13 (Histocompatibility Minor 13) to poor UM prognosis,^7^ but associations between UM and SASH3 (SAM And SH3 Domain Containing 3) or PLXNB1 (Plexin B1) have not been reported in the available literature to date. However, there does exist a wealth of convincing data correlating SASH3 and PLXNB1 activity with distinct clinical outcomes in several other cancers.^8^, ^9^, ^10^

**TABLE 1:** Overall survival probabilities associated with each hub gene, based on KM analysis.

Gene	Median OS of high expression group	Median OS of low expression group	*p*‐Value
*SASH3*	36.59	N/A	**1.08e‐03**
*PLXNB1*	N/A	31.07	**5.36e‐07**
*FAM107A*	36.59	51.98	3.73e‐01
*VSX2*	N/A	45.90	8.06e‐01
*TNKS2*	45.27	45.90	8.29e‐01
*HM13*	36.59	N/A	**1.12e‐04**

In this study, we describe novel master regulatory networks in the UM interactome. From these networks, we have further identified new genes associated with UM survival. Studies that further explore these relationships may realistically guide and predict immune therapy efforts. Potential limitations of our study include utilization of a single dataset and lack of specificity for local co‐expression, which is inherent to global gene co‐expression analytical techniques.

## CONFLICT OF INTEREST STATEMENT

Dr. Montanez‐Wiscovich serves as principal investigator for the CorEvitas registry sponsored by the National Psoraisis Foundation and the LITE study sponsored by the Patient Centered Outcomes Research Institute (PCORI). She also has an educational grant from Pfizer Global Medical Grants.

## FUNDING INFORMATION

The authors received no specific funding for this work.

## Data Availability

The data that support the findings of this study are available in the Broad GDAC Firehose portal at https://gdac.broadinstitute.org/.
